# Investigating the safety and efficacy of hematopoietic and mesenchymal stem cell transplantation for treatment of T1DM: a systematic review and meta-analysis

**DOI:** 10.1186/s13643-022-01950-3

**Published:** 2022-05-02

**Authors:** Sedigheh Madani, Mahdiyeh Amanzadi, Hamid Reza Aghayan, Aria Setudeh, Negar Rezaei, Mahtab Rouhifard, Bagher Larijani

**Affiliations:** 1 Diabetes Research Center, Endocrinology and Metabolism Clinical Sciences Institute, Tehran University of Medical Sciences, Tehran, Islamic Republic of Iran; 2 Cell Therapy and Regenerative Medicine Research Center, Endocrinology and Metabolism Molecular-Cellular Sciences Institute Tehran University of Medical Sciences, Tehran, Islamic Republic of Iran; 3 Children’s Medical Center, Tehran University of Medical Sciences, Tehran, Islamic Republic of Iran; 4 Non-Communicable Diseases Research Center, Endocrinology and Metabolism Population Sciences Institute, Tehran University of Medical Sciences, Tehran, Islamic Republic of Iran; 5 Endocrinology and Metabolism Research Center, Endocrinology and Metabolism Clinical Sciences Institute Tehran University of Medical Sciences, Tehran, Islamic Republic of Iran

**Keywords:** Clinical trial, Cell transplantation, Hematopoietic, Mesenchymal stem cell, Type 1 diabetes mellitus

## Abstract

**Background:**

Stem cell transplantation (SCT) has paved the way for treatment of autoimmune diseases. SCT has been investigated in type 1 diabetes mellitus (T1DM) as an autoimmune-based disorder, but previous studies have not presented a comprehensive view of its effect on treatment of T1DM.

**Methodology:**

After registration of the present systematic review and meta-analysis in the PROSPERO, a search was done according to the Cochrane guidelines for evaluation of clinical trials to find eligible clinical trials that investigated the effect of SCT on T1DM (based on ADA® diagnostic criteria) from PubMed, Web of science, Scopus, etc, as well as registries of clinical trials from January 1, 2000, to September 31, 2019. A search strategy was designed using MeSH and EM-tree terms. Primary outcome included the changes in the insulin total daily dose (TDD) (U/kg) level, and secondary outcomes included the changes in the HbA1c, c-peptide, and adjusted HbA1c levels. The *Q* Cochrane test and *I*^2^ statistic were performed to assess the heterogeneity and its severity in primary clinical trials. The Cochrane ROB was used to determine risk of bias, and Cochrane Handbook for Systematic Reviews of Interventions was used in the full text papers. The meta-analysis was accomplished in the STATA software, and the results were shown on their forest plots. Confounders were evaluated by the meta-regression test.

**Results:**

A total of 9452 studies were electronically screened, and 35 papers were included for data extraction. The results of this review study showed that 173 (26.5%) diabetic patients experienced insulin-free period (from 1 to 80 months), and 445 (68%) showed reduction in TDD of insulin after the SCT. Combination of hematopoietic stem cell (HSC) with mesenchymal stem cell (MSC) transplantation were significantly associated with improvement of the TDD (SMD: − 0.586, 95% CI: − 1.204/− 0.509, *I*^2^: 0%), HbA1c (SMD: − 0.736, 95% CI: − 1.107/− 0.365, *I*^2^: 0%), adjusted HbA1c (SMD: − 2.041, 95% CI: − 2.648/− 1.434, *I*^2^: 38.4%), and c-peptide (SMD: 1.917, 95% CI: 0.192/3.641, *I*^2^: 92.5%) on month 3 of follow-up, while its association had a growing trend from 3 to 12 months after the transplantation. Considering severe adverse events, HSC transplantation accompanied with conditioning could not be suggested as a safe treatment.

**Conclusion:**

Most of the clinical trials of SCT in T1DM were single arm. Although meta-analysis illustrated the SCT is associated with T1DM improvement, well-designed randomized clinical trials are needed to clarify its efficacy.

**Recommendation:**

Based on the results of this meta-analysis, the MSC and its combination with HSC could be considered as “Safe Cell” for SCT in T1DM. Furthermore, to evaluate the SCT efficacy, calculation of insulin TDD (U/kg/day), AUC of c-peptide, and adjusted HbA1c are highly recommended.

**Supplementary Information:**

The online version contains supplementary material available at 10.1186/s13643-022-01950-3.

## Background

T1DM imposes an increasing burden on the worlds̓ health system [1]. Insulin therapy is the gold standard treatment for T1DM, but it is invasive and complicated. Although the risk of overt nephropathy and severe retinopathy has been found to decrease through the tight glucose control by insulin therapy, noticeable complications are still observed in diabetic patients after 20–30 years of disease onset [2, 3]. This highlights the need for administration of a complementary treatment.

Based on the International Society for Pediatric and Adolescent Diabetes (ISPAD) Clinical Practice Consensus guidelines 2018, innovative treatment for insulin-dependent diabetes is as follows: glutamic acid decarboxylase (GAD)-alum vaccine, immune-modulating therapy, anti-thymocyte globulin (ATG), and granulocyte colony-stimulating factor (GCSF), and stem cell transplantation (SCT) [4].

Multi-potent stem cells have been transplanted for different kinds of autoimmune-based disorders resulting in some commercial products [5–8], but the use of SCT in T1DM is still in initial stages of safety and efficacy assessment. Since 2005, MSC and HSC have been transplanted to T1DM patients in some clinical trials [9–12], but lack of a consensus on their efficacy is obvious.

Efficacy of the SCT has been addressed in several papers [10–14]. It has been claimed that stem cells can improve the beta cell regeneration and treat long-term complications of the diabetes such as cardio-myopathy and neuropathy [15]. In addition, multi-potent stem cells could be easily isolated and expanded from different tissues with minimal immunogenicity, cost, and ethical concerns.

Several systematic reviews and meta-analyses have been done on the effect of SCT on T1DM but lack of some important items in each of them has caused a controversy in the results and made them incomplete to present a good portrait of previous interventional studies.

El-badawy et al. conducted a meta-analysis on SCT in 22 clinical trials in type 1 (16 trials) and type 2 (6 trials) diabetes. They used limited key words and searched different databases except for WOS and Scopus till August 2015. They demonstrated that MSC and CD34+ HSC transplantation were the most successful and effective approaches in patients with insulin-dependent diabetes as they resulted in occurrence of insulin-free period in 20–60% of the patients and 7–50% reduction in their insulin requirement [16].

Also, Gan et al. conducted a meta-analysis by missing important WOS database using MeSH words and some limited syntax to find the studies published till January 2018 and found 22 interventional papers that 8 (36%) of them were Chinese with limited electronic availability [17]. They illustrated that SCT is effective in reducing daily insulin requirement for a limited period of time.

Hwang et al. reviewed 6 clinical trials on T1DM and 10 studies on T2DM again by missing WOS database, using limited keywords till January 2018 [18]. They claimed that the SCT was not effective in treatment of T1DM while it was effective in T2DM.

Zhang et al. recently published an article which had reviewed 10 clinical trials on T1DM and 12 clinical trials on T2DM by missing Scopus and WOS databases till November 2018 [19]. They concluded that the HSC transplantation was more effective in improving diabetic parameter in comparison with MSC transplantation.

In this study, safety and efficacy of HSC and MSC transplantation for T1DM treatment were assessed through a systematic review of the interventional researches in accordance with the Methodological Expectations of Cochrane Intervention Reviews (MECIR) [20].

As mentioned in our systematic review protocol paper, “We believe that a standard systematic review and meta-analysis of primary clinical studies on the SCT in T1DM will help the clinicians and investigators to design more qualified and effective trials by choosing the best stem cells and the most suitable participants” [21].

## Materials and methods

### Protocol and registration

The PRISMA-P (Preferred Reporting Items for Systematic Review and Meta-Analysis Protocols) checklist was used to guide us in performing this systematic review [22]. The systematic review protocol was registered in the International Prospective Register of Systematic Reviews (PROSPERO) with registration number of CRD42016047176, and we also published this protocol [21]. The present systematic review manuscript was also checked using the PRISMA checklist [23].

### Eligibility criteria

Online eligible primary studies were collected according to their PICO-TS (Population, Intervention, Comparators, Outcome, Timing, and Study design) characteristics.

Inclusion criteria were as follows: P (population), all the researches on patients with T1DM diagnosed according to ADA® (American Diabetes Association; http://www.diabetes.org) diagnostic criteria regardless of their race, diabetes complications, age, and sex; I (intervention), HSC and/or MSC transplantation with any pre/post chemotherapy, any count, and from any route; C (comparison), clinical trials with control group or before-after comparison; O (outcome), for evaluating beta cell reserves and function, daily insulin dose (unit per kg) as the main primary outcome, and hemoglobin A1c (HbA1c) and c-peptide levels as secondary outcomes; T (timing), all the studies conducted between January 1, 2000, and September 31, 2019; and S (study design), all randomized and non-randomized clinical trials with no language limitation.

Exclusion criteria consisted of the following: P (population), patients with T1DM having monogenic diabetes marker; I (intervention), HSC, and/or MSC transplantation along with simultaneous Langerhans islet transplantation, T regulatory, or other kinds of SCT; O (outcome), researches that did not include assessment of daily insulin dosage as their outcome; T (timing), studies conducted before 2000 and after September 31 2019; and S (study design), all kinds of reviews, in vitro, and animal studies.

As mentioned in the protocol paper, the studies conducted between 2000 and 2017 were selected, but eligible studies published till 2019 were also added to have better coverage for analysis of the effect of SCT onT1DM.

### Resources

All the electronically published primary studies were extracted from important medical databases according to the Cochrane guidelines for evaluation of clinical trials [24] including PubMed, Web of Science, Scopus, and Embase; Cochrane Central Register of Control Trials (CENTRAL), Scientific Electronic Library Online (SCIELO), CINHal, Chinese citation index, and Indian citation index; and clinical trial registries of the World Health Organization (WHO) and countries with eligible researches (ClinicalTrials.gov, Chinese clinical trial registry, EU Clinical Trials Register (EU-CTR), Current Controlled Trials, or International Standard Randomized Controlled Trials Number (ISRCTN), Hong Kong Clinical Trials Register (HKUCTR), UMIN Clinical Trial Registry (UMIN-CTR), Iran Medex, Clinical Trials Registry of India, Brazilian Clinical Trials Registry (ReBecof), and Iranian Registry of Clinical Trials (IRCT)) and were also searched with no language restriction from January 1, 2000, to September 31, 2019.

### Search strategy used for PubMed and other resources

Keywords were selected after considering synonyms in EM-tree and MeSH databases as follows: “diabetes mellitus type 1,” “mesenchymal stem cell transplantation,” “hematopoietic stem cell transplantation,” and “stem cell transplantation.” PubMed database was searched with no language restriction from January 1, 2000, to September 31, 2019, and all the papers were cited in the EndNote version 7. The full text syntax is presented in Additional file 1. The final syntax was transformed for other databases. All the resources were investigated without publication or language restrictions. Conference papers in the SCOPUS, indexed in ProQuest database, WOS, and Google Scholar database were also searched electronically.

### Study selection

The first author removed the duplications after pooling all the outputs of the resources in the EndNote software then screened all the extracted papers using their titles and abstracts. Two independent reviewers evaluated screened papers for their eligibility and quality. Every primary study was assessed for its methodological quality by the Cochrane ROB to determine risk of bias for each study in order to classify them into high, fair, and poor quality [25]. Cochrane Handbook for Systematic Reviews of Interventions was used in selecting the full text papers [26]. Eligibility and quality of the studies were confirmed by discussion and consensus between the reviewers and under supervision of the SCT specialists and endocrinology subspecialists.

**Fig. 1:**
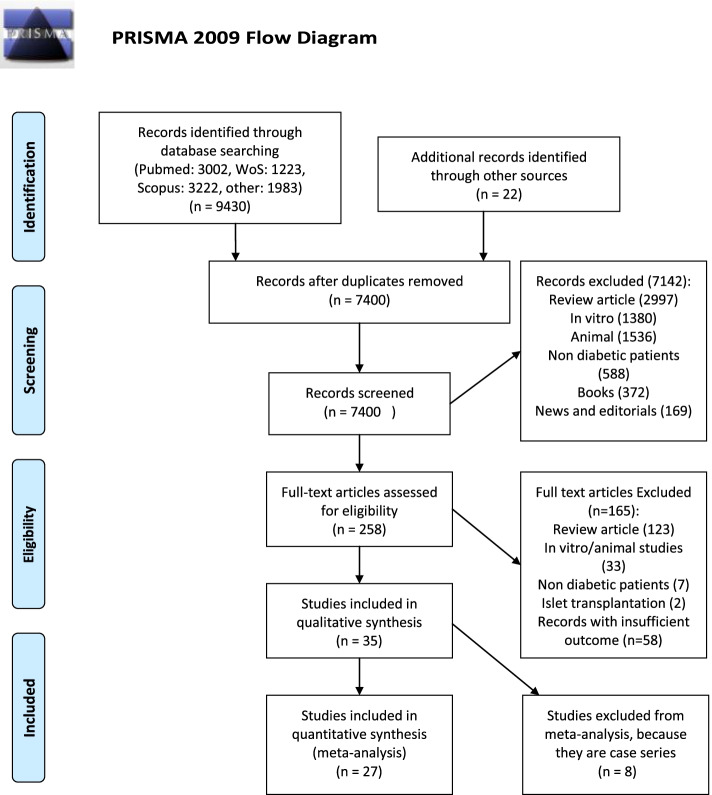
Flow diagram illustrating the identification, screening, and selection of the eligible clinical trials

A total of 9452 studies were electronically found while 2052 duplications were removed by EndNote software. Then, 7142 studies were omitted because there were reviews, in vitro or animal studies, news, editorials, or books (Fig. 1). Finally, the reviewers independently assessed 258 full texts to find eligible ones. Ninety-three papers met the inclusion criteria in the case of population, intervention, comparison, timing, and study design, but 58 of them were excluded because of incompatible main outcome (daily insulin requirement). At last, 35 papers were included for data extraction as listed in Table 1 and cited in the “References” section.

**Table 1:** Characteristic of 35 eligible papers included in this systematic review

Stem cell type	Author and year	type of study	Sample size	Mean age of patients (years)	Mean history of disease (years)	Mean dose of injected cells/kg	rout of injection	Number of insulin free	Mean follow-up period (months)	Reference
MSC	Carlsson 2015	RCT	18	24	0.04	2.75 × 10^6^	Peripheral vein	0	12	[27]
	Esfahani 2015	N-RCT	23	12.56	0.3	2 × 10^6^	Peripheral vein	2	12	[28]
	Hu 2013	RCT	29	17.6	0.42	2.6 × 10^7^	Peripheral vein	3	24	[29]
	Liu 2013	Case study	1	26	0.08	5.2 × 10^7^	Pancreatic dorsal artery	0	36	[30]
	Mesples 2013	Case study	3	7	0.15	181 × 10^6^	Intra-hepatic parenchyma	0	12	[31]
	Ulyanova 2019	Case study	5	30	Not mentioned	96 × 10^6^	Peripheral vein	0	3	[32]
HSC	Cantu-Rodriguez 2016	N-RCT	16	12	0.25	11.54 × 10^6^	Peripheral vein	7	34	[33]
	Ghodsi 2015	N-RCT	16	14.9	1	45 × 10^6^	Peripheral vein	3	12	[34]
	Couri 2009	N-RCT	23	18.4	0.05	3.0 × 10^6^	Peripheral vein	20	29.8	[35]
	D’Addio 2014	N-RCT	65	20.4	0.83	5.8 × 10^6^	Peripheral vein	46	48	[11]
	Ghodsi 2012	RCT	30	21.61	4.32	45 × 10^6^	Peripheral vein	0	12	[36]
	Gu 2012	N-RCT	28	17.6	0.22	Not mentioned	Peripheral vein	20	23	[37]
	Gu 2014	N-RCT	42	8.04	0.25	Not mentioned	Peripheral vein	3	29	[38]
	Gu 2017	N-RCT	40	17.9	0.16	Not mentioned	Peripheral vein	14	48	[39]
	Haller 2011	N-RCT	24	5.1	0.25	Not mentioned	Peripheral vein	0	24	[40]
	Leal 2012	N-RCT	13	16	0.1	Not mentioned	Peripheral vein	4	24	[41]
	Li 2012	N-RCT	13	14.1	0.41	2 × 10^6^	Peripheral vein	3	42.38	[42]
	Mesples 2007	N-RCT	12	36	13	1 × 10^6^	Pancreatic circulation	4	6	[43]
	Shen 2012	case study	1	21	0	5.8 × 10^6^	Peripheral vein	1	70	[44]
	Snarski 2009	case study	1	28	0.07	3.03 × 10^6^	Peripheral vein	1	3	[45]
	Snarski 2011	N-RCT	8	25.8	0.16	3 × 10^6^	Peripheral vein	8	7	[46]
	Snarski 2016	N-RCT	24	26.5	0.17	4.19 × 10^6^	Peripheral vein	20	52	[47]
	Tootee 2015	N-RCT	72	17.52	0.43	45 × 10^6^	Peripheral vein	0	12	[48]
	Voltarelli 2007	N-RCT	15	19.2	0.05	11.0 × 10^6^	Peripheral vein	8	18.8	[49]
	Xiang 2016	N-RCT	112	15.58	0.1	3 × 10^6^	Peripheral vein	0	15.13	[50]
	Ye 2017	N-RCT	18	18.86	0.42	2 × 10^5^	Peripheral vein	0	12	[51]
	Zhang 2012	N-RCT	9	18.5	0.16	2.63 × 10^6^	Peripheral vein	6	12	[52]
Combined SC	Cai 2016	RCT	63	19.33	9.24	106.78 × 10^6^ HSC 1.1 × 10^6^ MSC	Dorsal pancreatic artery	0	12	[53]
	Dave 2013	Case study	1	30	15	34 × 10^6^ HSC 5.4 × 10^4^ MSC	Portal and thymus circulation	0	13	[54]
	Dave 2014	Case study	2	5,9	7,2	51.7 × 10^6^ HSC 1.66 × 10^4^ MSC	portal and thymus circulation	0	18,24	[55]
	Dave 2015	N-RCT	10	20.2	8.1	60.55 × 10^6^ HSC 2.7 × 10^6^ MSC	Portal and thymus circulation	0	31.71	[56]
	Thakkar 2015	N-RCT	20	19.95	8	2.21 × 10^4^HSC 2.65 × 10^4^ MSC	Portal system and thymus artery	0	54.24	[57]
	Trivedi 2008	N-RCT	5	20.2	4.5	3.15 × 10^6^ (HSC + MSC)	Portal circulation	0	2.9	[58]
	Trivedi 2011	N-RCT	2	18	11	3.8 × 10^6^ (HSC + MSC)	Portal circulation	0	27	[59]
	Vanikar 2010	N-RCT	11	21.1	8.2	26.96 × 10^8^ HSC 11.55 × 10^7^ MSC	Portal circulation	0	7.3	[60]

The eligible papers were evaluated in two main sections; safety and efficacy

### Data extraction and management

The reviewers independently extracted the information of the studies such as their titles, first authors̓ name, publication year, number of patients in the intervention and control groups, mean interval between diabetes diagnosis and intervention, positive history of DKA, mean age of the participants, auto antibodies titer in the diabetes, mean dose of transplanted stem cell, number of insulin-free patients, adverse effect of intervention with its severity, and baseline and follow-up amount of diabetes parameters (c-peptide, HbA1c, and TDD of insulin) with follow-up period in 3, 6, and 12 months. Consensus and/or attitude of the specialists assisted them to decide in the case of conflicts.

Also, the reviewers assessed the adverse event of intervention in each eligible study according to the Common Terminology Criteria for Adverse Events (CTCAE), Version 5.0 [61] in two steps. First, they extracted the data based on the kind of the adverse events in each clinical trial, and then, the adverse events of the same kind were pooled to illustrate their incidence.

In the case of safety, our endocrinologists discussed to determine whether the adverse event is a consequence of the intervention or it is one of the complications of the T1DM and routine insulin therapy. According to their opinions, some of the mentioned adverse events were excluded in the eligible trials, including hypoglycemia because it could occur due to insulin therapy and thyroid autoimmune disorders along with T1DM without any intervention.

The quality of the research was assessed by the Cochrane’s tool to determine risk of bias of randomized controlled trials [24], and thresholds for converting the Cochrane’s risk of bias tool was used to apply the AHRQ (the Agency for Healthcare Research and Quality) standards and categorize the studies into poor, fair, and good quality. Almost all the studies had poor quality in their design except one [29] that had a good quality.

An email was sent to corresponding authors of the studies to recover the missing data, and if it was not possible to receive the main outcome measures, then that paper was omitted. In addition, in the case of different follow-up intervals, studies with 3, 6, and 12 months of follow-up were included, which was the same in most of the studies. All the process of data extraction and meta-analysis was completed independently by 2 reviewers.

### Heterogeneity and risk of bias assessment

The *Q* Cochrane test and *I*^2^ statistic were performed to assess the heterogeneity and its severity in primary clinical trials. The *P* value less than 0.05 was considered as statistically significant but the endocrinologists̓ attitude was also asked to interpret the results. The reviewers evaluated the methodological quality of eligible papers by the Cochrane ROB [25].

Some factors may affect heterogeneity of the studies, including the design of primary study, kind of stem cell, history of DKA, age of the participants, and the time interval between diagnosis and intervention [19]. These confounders also assessed by meta-regression analysis.

Publication bias was assessed using the funnel plot and Begg’s and Egger’s regression test, and the results were checked by the Trim-and-Fill test.

### Data synthesis and meta-analysis

The eligible papers were meta-analyzed in the efficacy section. The case studies were evaluated in the systematic review, but they were omitted in the meta-analysis. We chose standardized mean difference as the effect size, and eligible researches were meta-analyzed in two steps. First, the randomized and non-randomized papers that had control group were meta-analyzed. Then, clinical trials with or without control group were meta-analyzed. In this meta-analysis, intervention groups were compared with their own control group. In the second meta-analysis, only the intervention group of each research was entered, and their outcomes were compared. Subgroup analysis was performed for type of stem cells, different follow-up periods (3, 6, and 12 months), and different outcomes.

The meta-analysis was completed on STATA software version 14, and the results were shown on their forest plot.

In addition, confounders were evaluated by meta-regression test, for mean age of the patients, history of DKA, and mean interval between diagnosis and intervention.

## Results

A total of 9452 studies were electronically found, and finally 35 papers were included for data extraction as listed in Table 1 and cited in the “References” section (Fig. 1). All interventional studies except case studies with 5 and less patients were included in the systematic review. Therefore, the sample size of eligible studies for meta-analysis ranged from 8 to 112; totally, 754 subjects were meta-analyzed (655 cases and 99 controls). The age range of the participants was 1.5 to 60 years old.

Table 1 shows the eligible studies divided into the three groups according to the type of transplanted stem cell.

The eligible papers were evaluated in two main sections: safety and efficacy.

### SCT safety

**Fig. 2:**
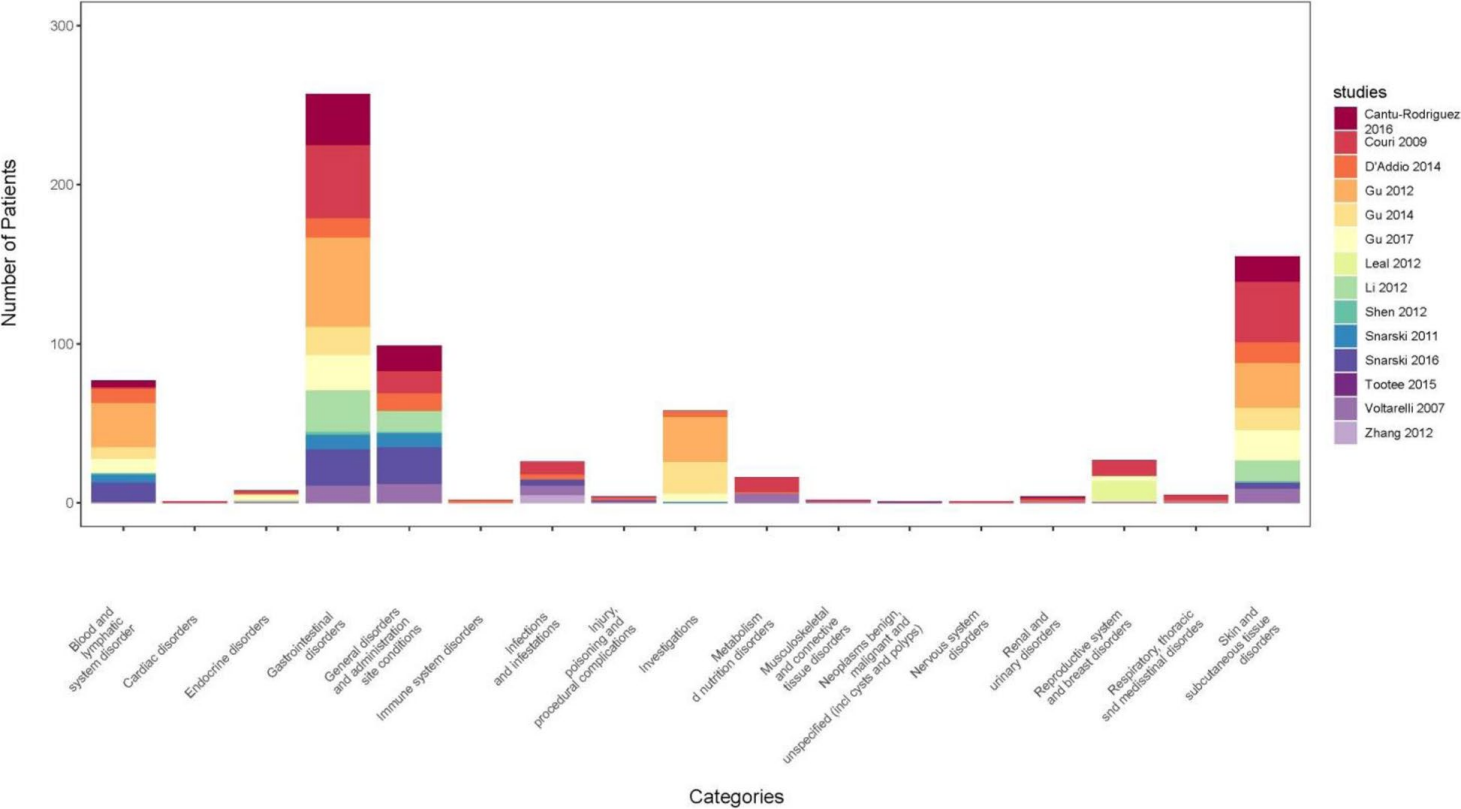
Number of each adverse effect reported in each article according to the system organ class in the CTCAE

All the 6 clinical trials of MSC transplantation were reported to have no adverse events. Eligible clinical trials in which the HSC and MSC were transplanted were also mentioned to have no prominent adverse event. The main side effects were reported in the trials of the HSC transplantation with conditioning. Seven of these clinical trials were not found to have any side effect, but in 14 clinical trials, prominent adverse events were reported, which are summarized in Fig. 2 and Table 2 according to the side effect category of the CTCAE.

**Table 2:** The number of patients suffering from each side effect in each system organ class according to the CTCAE. Articles reported each side effect was also mentioned in references column. (SOC, system organ class according to CTCAE; Terms, CTCAE terms for each sign or symptom, grading each side effect performed according to the CTCEA scoring chart)

Categories	Disease	Grade 1	Grade 2	Grade 3	Grade 4	Grade 5	Total	References
Blood and lymphatic system disorders	Febrile neutropenia			70			70	[11, 33, 37, 39, 46, 47]
	White blood cell decreased (leukopenia)	10					10	[35, 38, 44, 49]
Cardiac disorders	Sinus bradycardia	1					1	[35]
Endocrine disorders	Hyperthyroidism (Graves)		3				3	[35, 39]
	Hypothyroidism		5				5	[35, 37, 39, 42, 49]
Gastrointestinal disorders	Diarrhea		15				15	[11, 35, 38, 46, 49]
	Dyspepsia		9				9	[11]
	Mucositis oral		2	1			3	[11, 35, 49]
	Nausea	3	126				129	[11, 33, 35, 37, 39, 42, 44, 46, 47, 49]
	Vomiting	3	110				113	[33, 35, 37–39, 42, 44, 49]
General disorders and administration site conditions	Edema limbs		3				3	[11, 46]
	Fever	60	34				94	[11, 33, 35, 42, 44, 46, 47, 49]
	Generalized edema (fluid overload)	3					3	[35, 49]
Immune system disorders	Allergic reaction			2			2	[11]
Infections and infestations	Bacteremia		5				5	[39[71]
	Catheter-related infection		4				4	[35, 49]
	Folliculitis	1					1	[36]
	Herpes simplex reactivation		2				2	[35, 49]
	Lung infection (pneumonia)			3			3	[35, 49]
	Sepsis				4	2	6	[11, 47]
	Sinusitis		2				2	[35, 49]
	Skin infection (pyoderma)			2			2	[35, 49]
	Vaginal infection (vulvovaginal candidiasis)		1				1	[52]
Injury, poisoning, and procedural complications	Vascular access complication		2				2	[11, 47]
	Venous injury		2				2	[35, 49]
Investigations	Activated partial thromboplastin time prolonged (coagulopathy)		1				1	[11]
	Alanine aminotransferase increased	1					1	[46]
	Bone marrow hypocellular (BM suppression)		15	15	5		35	[11, 37, 39]
	Neutrophil count decreased (neutropenia)	7					7	[37, 38]
	Weight loss	7	7				14	[38]
Metabolism and nutrition disorders	Anorexia	13					13	[35, 49]
	hypokalemia		2				2	[35, 49]
	Metabolism and nutrition disorders—other, specify (nutrition support)			1			1	[11]
Musculoskeletal and connective tissue disorders	Rhabdomyolysis		2				2	[35, 49]
Neoplasms benign, malignant and unspecified (incl. cysts and polyps)	Neoplasms benign				1		1	[48]
Nervous system disorders	Headache		1				1	[36]
Renal and urinary disorders	Cystitis non-infective	1	1				2	[33]
	Dysuria	1					1	[35, 49]
	hematuria		1				1	[11]
Reproductive system and breast disorders	Irregular menstruation		3				3	[39]
	Oligospermia	22					22	[41]
	Reproductive system—other, specify (hypogonadism)		2				2	[35, 49]
Respiratory, thoracic, and mediastinal disorders	Epistaxis		3				3	[35, 49]
	Pharyngeal mucositis	1					1	[35]
	Pneumothorax		1				1	[11]
Skin and subcutaneous tissue disorders	Alopecia	3	128				131	[11, 33, 35, 37–39, 42, 44]
	Purpura (hemorrhagic rash)			1			1	[47]
	Rash maculopapular (skin rash)	20	2	2			24	[11, 35, 47]
	Urticaria	2	3				5	[35, 49]

These side effects were also scored according to the CTCAE in 5 grades of severity as illustrated in Table 2. Alopecia, nausea, and vomiting were the most frequent side effects, but mortality was the most important side effect occurred in 2 patients due to the sepsis. Life-threatening sepsis and bone marrow aplasia (for more than 2 weeks) were respectively reported in 4 and 5 patients after the non-myeloablative (NMA) conditioning.

Also, a case of benign transitional meningioma was reported by Nasli et al. after fetal HSC transplantation without conditioning. They suggested that the tumor cells did not have the genetic characteristics of the patients’ cells, but they could not compare the genetic characteristics of the tumor cells with the transplanted cells because there was not any retained sample of transplanted fetal HSCs at the time of the meningioma diagnosis.

### SCT efficacy

Most of the eligible papers were single-arm clinical trials; hence, meta-analysis was performed in two steps. At first, 7 eligible clinical trials consisting of the control group (randomized or non-randomized) were meta-analyzed. The results showed considerable effect of SCT on the reduction of insulin TDD and c-peptide at 12 months after intervention while comparing SMD as effect size between the intervention and control groups. Although HbA1c decreased, its reduction was not significant (Table 3).

**Table 3:** The effect of SCT on the diabetes parameters (insulin TDD, HbA1c, c-peptide, and adjusted HbA1c) in different follow-up periods

Outcome		month	No. of studies	No. of patients	Standard mean difference	95% confidence interval	*P* value	Heterogeneity
Meta-analysis Step 1	Insulin TDD (U/kg/day)	12	7	171	− 1.741	− 3.034/− 0.448	0.008	94.5%
	HbA1c (%)	12	6	153	− 0.169	− 0.697/0.358	0.530	84.6%
	c-Peptide (ng/ml)	12	7	171	0.413	0.097/0.728	0.010	54.5%
Meta-analysis Step 2	Insulin TDD (U/kg/day)	3	20	381	− 1.245	− 1.727/− 0.764	0.000	88.3%
		6	18	343	− 1.483	− 2.049/− 0.918	0.000	90.2%
		12	21	436	− 2.154	− 2.975/− 1.332	0.000	95.7%
	HbA1c (%)	3	21	388	− 1.283	− 1.722/− 0.795	0.000	88.8%
		6	21	446	− 1.624	− 2.334/− 0.914	0.000	94.9%
		12	24	598	− 1.832	− 2.427/-1.237	0.000	94.7%
	c-Peptide (ng/ml)	3	15	305	0.369	0.135/0.872	0.003	85.1%
		6	16	328	0.757	0.288/1.226	0.002	87.0%
		12	21	550	1.146	0.534/1.759	0.000	94.7%
	Adjusted HbA1c	3	16	318	− 2.406	− 3.415/− 1.397	0.000	95.7%
		6	17	367	− 3.883	− 5.479/− 2.287	0.000	95.8%
		12	18	432	− 3.828	− 5.538/− 2.118	0.000	98.3%

In the second step of meta-analysis, 27 eligible papers were studied, while 7 of them were case studies. As shown in Table 3, the results demonstrated that SCT is associated with the reduction of insulin TTD, HbA1clevels, increment of c-peptide, and adjusted HbA1c levels in 3, 6, and 12 months follow-up. Considering the high level of studies’ heterogeneity, these results seem to be inconclusive.

Interestingly, it was found that the SCT had a growing effect on the diabetes in most of the mentioned parameters from 3 to 12 months after the transplantation while comparing SMD as effect size. Adjusted HbA1c increased until 6 months after SCT then, it decreased a little in the next 6 months. None of these trends was statistically significant.

SCT had been performed in 655 T1DM patients. Totally, 173 (26.4%) patients experienced insulin-free period (from 1 to 80 months) and 449 (68.5%) of the participants showed a reduction in the TDD of insulin. The SCT efficacy was also checked after classifying the studies according to the type of transplanted stem cells.

DKA history, age of the participants, and interval between diagnosis and intervention were not significantly important to confound the effect of SCT on daily insulin requirement, HbA1c, adjusted HbA1c, or c-peptide levels as analyzed by the meta-regression.

**Fig. 3:**
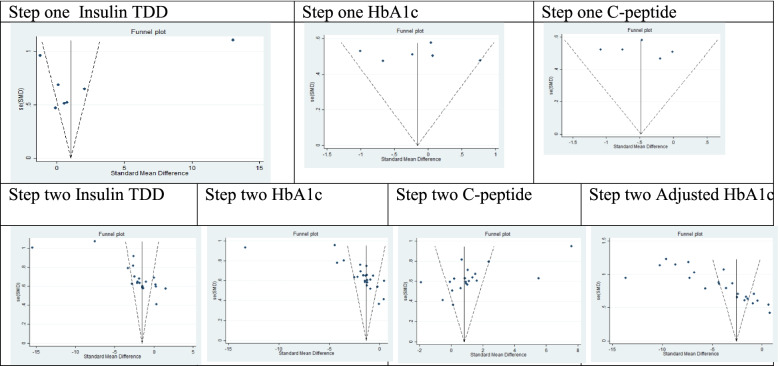
Publication bias assessment according to the funnel plots for step 1 meta-analysis and month 12 of follow-up in step 2 meta-analyses, which is presented in Table 3

Publication bias was assessed using the funnel plot and Begg’s and Egger’s regression test for forest plots of the total SCT meta-analysis (Table 3). The publication bias analysis showed a significant bias and the results were shown in Fig. 3.

**Fig. 4:**
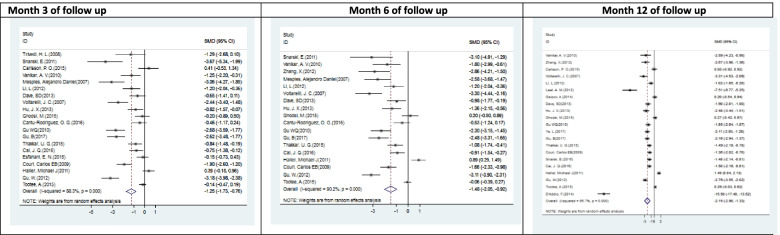
Forest plots with the corresponding 95% CIs for the correlation between insulin TDD (U/kg) and SCT in T1DM (meta-analysis of Table 3)

**Fig. 5:**
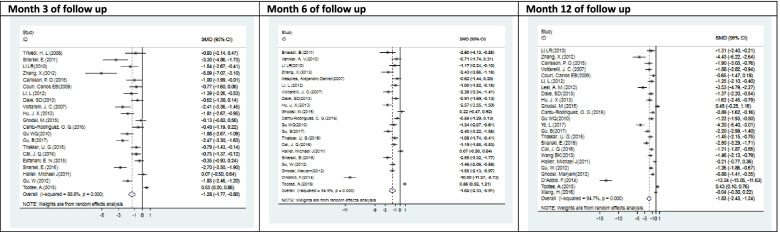
Forest plots with the corresponding 95% CIs for the correlation between the HbA1c (%) and SCT in T1DM (meta-analysis of Table 3)

**Fig. 6:**
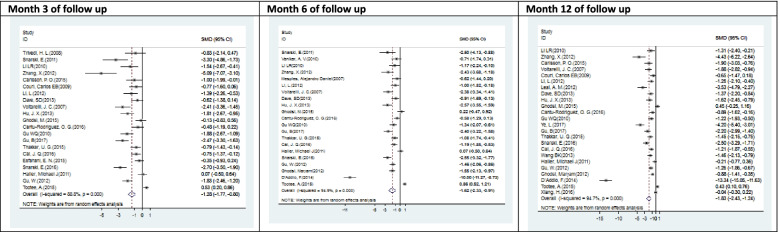
Forest plots with the corresponding 95% CIs for the correlation between the c-peptide (ng/ml) and SCT in T1DM (meta-analysis of Table 3)

**Fig. 7:**
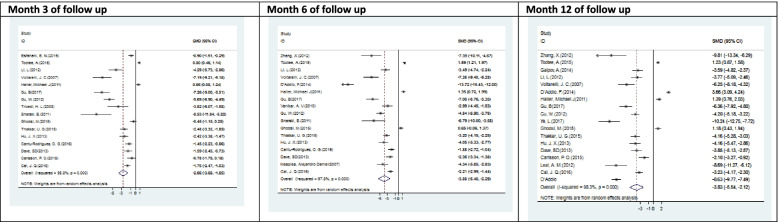
Forest plots with the corresponding 95% CIs for the correlation between adjusted HbA1c (%) and SCT in T1DM (meta-analysis of Table 3)

Figs. 4, 5, 6, and 7 showed all meta-analysis’ forest plots and compared them according to the outcomes, including insulin TDD, HbA1c, C-peptide, and adjusted HbA1c.

### Efficacy of the MSC transplantation

Six of the included researches investigated the MSC transplantation. Totally, they investigated 74 diabetic patients, while 24 of them were in the control group. Most of these patients received MSCs through peripheral vein injection, while in one patient, dorsal pancreatic artery was used [30], and in two patients, liver puncture was selected as the route of administration [31]. Nasli et al. administered two injections at 3-month intervals [28], so the second intervention was omitted and the effect of first injection after 3 months of follow-up was included. Insulin injection was the only standard treatment received by the participants in the MSC therapy subgroup.

Totally, 5 (10%) patients experienced insulin-free period (from 1 to 24 months), and 23 (46%) of the participants showed a reduction in TDD of insulin. Subgroup meta-analysis showed that MSC therapy is efficient in treatment of T1DM.

Data was not enough for meta-analysis in this subgroup because just 2 or 3 researches had data for 3 and 12 months after MSC therapy. The MSC therapy had a considerably growing association with all the mentioned diabetic parameters from 3 to 12 months after the transplantation while comparing SMD as effect size showed that these trends were not statistically significant. Heterogeneity in the MSC therapy subgroup ranged between low (00.0%) and high (82%) in some points (Table 4).

**Table 4:** MSC effect on diabetes parameters (Insulin TDD, HbA1c, C-peptide, and Adjusted HbA1c) in different follow-up periods

Outcome		Month	Standard mean difference	95% confidence interval	*P* value	Heterogeneity
MSC therapy effect	Insulin TDD (U/kg/day)	3	− 0.226	− 0.851/0.398	0.477	53.1%
		6^a^	–	–	–	–
		12	− 1.235	− 3.663/1.193	0.319	82.4%
	HbA1c (%)	3	− 1.011	− 1.919/− 0.103	0.029	74.2%
		6^a^	–	–	–	–
		12	− 1.715	− 2.386/− 1.045	0.000	00.0%
	c-Peptide (ng/ml)	3	0.243	− 0.163/0.649	0.240	00.0%
		6^a^	–	–	–	–
		12	1.234	0.612/1.856	0.000	00.0%
	Adjusted HbA1c	3	− 1.337	− 2.296/− 0.377	0.006	74.8%
		6^a^	–	–	–	–
		12	− 3.119	− 5.132/− 1.087	0.003	81.2%

^a^Represents scant data for meta-analysis (only data of one study was available)

### Efficacy of the HSC transplantation

HSC therapy was administered in 21 of the included researches. In these researches, 491 patients received transplantation with HSC, and their results were compared with 75 control participants. HSC transplantation was conducted in two main approaches based on being autologous or allogenic.

Most of the studies in this subgroup had isolated the mobilized autologous HSC from peripheral circulation by leukapheresis after administration of granulocyte colony-stimulating factor (GCSF) and cyclophosphamide. They also performed NMA conditioning for a week between harvesting the HSC and transplantation.

In the second group of clinical trials, allogenic fetal liver derived HSCs were administered. No conditioning was performed in this group.

In both approaches, HSC was injected in peripheral vein, except in 12 participants who received cells from pancreatic circulation [43]. All the participants received their daily insulin along with the interventions if needed.

Insulin-free period was experienced by 168 (34.2%) patients (from 1 to 80 months), and 332 (67.6%) of them just showed some reduction in TDD of insulin. Subgroup meta-analysis showed that HSC therapy could be associated with an improvement of beta cell function.

The HSC therapy had a growing effect on most of the mentioned parameters from 3 to 12 months after the transplantation while comparing SMD as effect size, but none of these trends were statistically significant (Table 5). Adjusted HbA1c increased until 6 months after SCT; then, it decreased in the next 6 months.

**Table 5:** The effect of HSC transplantation on the diabetes parameters (insulin TDD, HbA1c, C-peptide, and adjusted HbA1c) in different follow-up periods

Outcome		Month	Standard mean difference	95% confidence interval	*P* value	Heterogeneity
HSC therapy effect	Insulin TDD (U/kg/day)	3	− 1.683	− 2.451/− 0.915	0.000	92.5%
		6	− 1.620	− 2.401/− 0.839	0.000	92.9%
		12	− 2.408	− 3.494/− 1.322	0.000	96.8%
	HbA1c (%)	3	− 1.542	− 2.264/− 0.819	0.000	92.4%
		6	− 1.744	− 2.652/− 0.836	0.000	96.1%
		12	− 1.943	− 2.660/− 1.226	0.000	95.7%
	c-Peptide (ng/ml)	3	0.369	− 0.163/0.649	0.151	81.1%
		6	0.452	0.038/0.867	0.032	79.3%
		12	0.813	0.118/1.509	0.022	95.3%
	Adjusted HbA1c	3	− 2.951	− 4.689/− 1.214	0.001	97.4%
		6	− 4.316	− 6.465/− 2.166	0.000	98.3%
		12	− 3.972	− 6.069/− 1.875	0.000	98.5%

Heterogeneity in the HSC therapy subgroup was high, so the studies with no chemotherapy were omitted to make remaining ones similar, but heterogeneity remained high again. These high levels of heterogeneity suggest that we could not consider these results as powerful results.

### Efficacy of the combined MSC and HSC transplantation

In the 8 eligible studies, HSC and MSC were co-transplanted in 114 diabetic patients. Cai et al. transplanted autologous peripheral blood HSC without conditioning along with umbilical cord MSC in 42 patients through dorsal pancreatic artery [53]. In other trials, NMA conditioning was done, and HSCs were transplanted along with adipose tissue MSCs in 49 patients via portal system. All the participants received their daily insulin simultaneously with the trials if needed.

Insulin-free period was experienced by none of the patients, and 92 (80.7%) of the participants showed a reduction in TDD of insulin. Subgroup meta-analysis showed that combined SCT is efficient in some of T1DM parameters.

Comparing SMD as effect size revealed that co-administration of HSC and MSC significantly improved the daily insulin requirement (SMD: − 0.586, 95% CI: − 1.204/− 0.509, *I*^2^: 0%), HbA1c (SMD: − 0.736, 95% CI: − 1.107/− 0.365, *I*^2^: 0%), adjusted HbA1c (SMD: − 2.041, 95% CI: − 2.648/− 1.434, *I*^2^: 38.4%), and c-peptide (SMD: 1.917, 95% CI: 0.192/3.641, *I*^2^: 92.5%) on month 3 of follow-up, while its effect had a growing trend from 3 to 12 months after the transplantation (Table 6). The combined SCT had an enhancing direct relation with most of the mentioned parameters from 3 to 12 months after the transplantation while comparing SMD as effect size. The c-peptide level only increased until 6 months; then, it decreased in the next 6 months of follow-up. These trends were not statistically significant except for adjusted HbA1c. The adjusted HbA1c significantly (*P* value: 0.044) improved from month 3 to month 12 of transplantation. Interestingly, minimum heterogeneity (0%) was observed in the combined SCT subgroup except in meta-analysis of c-peptide. So, we could not rely on the c-peptide changes after combined MSC and HSC transplantation as a powerful result.

**Table 6:** Effect of combined SCT on the diabetes parameters (insulin TDD, HbA1c, C-peptide, and adjusted HbA1c) in different follow-up periods

Outcome		Month	Standard mean difference	95% confidence interval	*P* value	Heterogeneity
Combined SCT effect	Insulin TDD (U/kg/day)	3	− 0.586	− 1.204/− 0.509	0.000	00.0%
		6	− 1.067	− 1.443/− 0.690	0.000	00.0%
		12	− 1.652	− 2.070/− 1.234	0.000	00.0%
	HbA1c (%)	3	− 0.736	− 1.107/− 0.365	0.000	00.0%
		6	− 1.027	− 1.401/− 0.653	0.000	00.0%
		12	− 1.334	− 1.749/− 0.918	0.000	00.0%
	c-Peptide (ng/ml)	3	1.917	− 0.192/3.641	0.029	92.5%
		6	3.025	− 0.773/6.822	0.118	96.5%
		12	2.544	0.476/4.612	0.016	93.9%
	Adjusted HbA1c	3	− 2.041	− 2.648/− 1.434	0.002	38.4%
		6	− 2.590	− 3.074/− 2.107	0.000	00.0%
		12	− 3.668	− 4.293/− 3.042	0.000	00.0%

## Discussion

According to the results of this meta-analysis, MSC, HSC, and their co-transplantation are significantly associated with T1DM improvement. From 50 patients who received MSCs transplantation, 5 patients experienced insulin-free period, and 23 participants showed reduction in TDD of insulin. No adverse effect was observed in these patients. HSCs in combination with NMA conditioning were conducted in 491 patients. Insulin-free period was experienced by 168 patients, and 332 of them showed some reduction in TDD of insulin. Several adverse events consisting of life-threatening complications and death were seen in this group.

In contrast to HSC transplantation with NMA conditioning, co-transplantation of HSC and MSC had no prominent side effect. From 114 patients who co-transplanted with HSC and MSC, no one experienced insulin-free period, but in 92 of them, reduction in TDD of insulin was observed.

Meta-analysis by El-badawy et al. [4] and Gan et al. [17] also demonstrated that MSC and CD34+ HSC were the most successful and effective SCT approaches in the patients with insulin-dependent diabetes, while Hwang et al. [18] claimed that the SCT was not effective in treatment of T1DM.

Although our total and subgroups meta-analysis showed a trend of SCT efficacy from 3 to 12 months after the transplantation, these trends were not statistically significant except for the adjusted HbA1c. The adjusted HbA1c in MSC and HSC co-transplantation group significantly improved from month 3 to month 12 of transplantation.

Discontinuation of the insulin injection was found as the most important parameter for improvement of quality of life in the diabetic patients, so it was mentioned as the main outcome. The results of this systematic review showed that 173 (26.4%) patients experienced insulin-free period (from 1 to 80 months), and 449 (68.5%) of the participants showed reduction in TDD of insulin. Most of the SCT clinical trials in T1DM missed control group and had a high level of heterogeneity, and it made the results inconclusive. Although it seems that the SCT is associated with T1DM improvement, some points remain to be clarified in the future studies.

### C-peptide

Most of the researchers had measured the fasting c-peptide, so they have missed to measure the postprandial beta cell function, which could be evaluated by area under the curves (AUCs) of peak c-peptide and c-peptide. To evaluate the beta cell function, AUC for c-peptide instead of fasting c-peptide level has been recommended by the ISPAD Clinical Practice Consensus Guidelines 2018 [4, 62, 63].

### Adjusted HbA1c and DKA

Some clinical trials have focused on DKA and claimed that history of DKA would reduce efficacy of the SCT for T1DM treatment [37]. It has been concluded that the patients with DKA have less beta cell reservation for regeneration. In this study, it was found that history of DKA did not significantly confound the SCT efficacy as analyzed by meta-regression.

We found some reasons for this controversy. DKA is a sign of insulinopenia in which beta cells could not produce enough insulin to maintain the balance between insulin and its counter-regulatory hormones. DKA arises without any stress when number of beta cell declines to 10% of their primary count. The point is that DKA can also occur when beta cell reservation is between 30 and 40%, and the patient experiences a major stress [64], so it would not be a good parameter to predict the SCT efficacy.

Some previous studies have discussed about *β*-score to determine the beta cell reservation in order to measure the islet transplantation and SCT efficacy in treatment of T1DM [50, 65]. *β*-score is measured based on fasting plasma glucose (mmol/l), daily insulin consumption (unit/kg), HbA1c (%), and peak/fasting c-peptide (nmol/l). It ranges between 0 and 2, while score 2 represents good beta cell function and normal glucose homeostasis, score 1 shows partial glucose homeostasis, and score 0 corresponds with glucose imbalance. If *β*-score is evaluated in the future SCT trials, efficacy measure for this innovative treatment will be more reliable.

Adjusted HbA1c which has been previously studied to predict the honey moon period is another factor estimating the beta cell reservation [66, 67]. Adjusted HbA1c determines the partial remission period after starting the insulin therapy that may occur in some patients with T1DM [68]. In this study, adjusted HbA1c was calculated in eligible trials for the first time to evaluate the SCT effect on T1DM.

### Heterogeneity

In the present study, heterogeneity reduced when the studies were classified according to type of transplanted stem cell. However, the heterogeneity in the HSC subgroup was high again, while homogeneity was observed in the MSC and combined SCT subgroups. Heterogeneity in the HSC subgroup may be due to different follow-up periods, whereas some of HSC trials followed up their participants for 5 years, some followed up just for 3 months. Also, heterogeneity may result from the patient’s nutrition, exercise, or weight and stem cell dose.

AHRQ standard was applied and the studies were categorized into poor, fair, and good quality. Almost all the studies had poor quality in their design except one [29] that had a good quality. Poor design of primary studies may be the other cause of heterogeneity in our meta-analysis.

It was also tried to contact with two research groups whose papers had some points for clarification, but they did not respond to our email for two times.

### Transplantation routes

In reviewed studies, stem cell transplantation had been administered by two main routes, including targeted and untargeted transplantation. Targeted SCT transmits the stem cells directly to the damaged organ and seems to be more effective and also more invasive. Untargeted SCT supplies stem cells from peripheral circulation so that stem cells should pass lung capillary system to reach damaged organ. Some investigators believe that HSC and MSC have anti-inflammatory and immune-modulatory characteristics in a way that the stem cells find their niche in the damaged organ [69, 70]. Thus, the route of stem cell transplantation should be considered as one of the main factors to design a clinical trial for SCT.

In screening and full text review, one clinical trial registration number was found in several papers [33, 57, 71, 72]. These papers could be written about different phases of follow-up or presenting the primary outcomes regarding the effect of SCT on diabetes complications.

### Safety of SCT

The basic complications of T1DM and insulin therapy should be determined to distinguish between the adverse events of the intervention. Adhering to Good Clinical Practice (GCP) with randomization and inclusion of a control group make the segregation of intervention related adverse events easier. Unfortunately, almost all the SCT clinical trials for treatment of T1DM had poor quality, no randomization was performed, and most of them had no control group to compare the outcomes and adverse events. Therefore, there were some conflicts in judging about the SCT side effects in these trials, and it was decided to omit the hypoglycemia because it could occur due to the insulin therapy and thyroid autoimmune disorder along with T1DM without any intervention.

HSC transplantation with NMA conditioning resulted in the immune-suppression and made the patients prone to affection with the opportunistic infections. As a rule, the scientists prefer to utilize more safe interventions to further guarantee the health of the participants. In this regard, major side effects have been reported for HSC transplantation with the protocol consisting of the chemotherapy. Considering these facts, it is suggested to focus on the MSC therapy in T1DM instead of HSC therapy. Moreover, previous studies have shown the efficacy of MSCs transplantation in treatment of patients with autoimmune disorders [73–75].

### Recommendation for future clinical studies

Most of the eligible SCT studies in T1DM were single-arm clinical trials, while an informative clinical trial should include a reliable control group. Herein, the studies were meta-analyzed in two steps, and the results illustrated the effect of the SCT on reduction of insulin TDD and the increase in the c-peptide level, 12 months after the transplantation (Table 3).

Since administration of the SCT in T1DM has not been approved by regulatory agencies, most of the studies were conducted by the geographically scattered institutes causing the lack of well-designed researches.

SCT could be potentially effective in treatment of T1DM, and it is recommended to conduct further well-designed researches to provide a real perspective of this new intervention. Furthermore, we propose to study the SCT effect by measuring the insulin TTD, HbA1c, c-peptide’s fasting and postprandial levels, and T1DM auto antibodies (zinc transporter 8, glutamic acid decarboxylase (GDA), islet cell cytoplasmic auto-antibodies (ICA) in the beta cell, and insulin auto antibodies to protein tyrosine phosphatase) before and 1, 3, 6, 12, 18, and 24 months after the transplantation and then in yearly follow-up to determine its efficacy on the mentioned parameters.

As insulin TDD, HbA1c, and c-peptide levels vary naturally in the first 2 years of T1DM onset and beta cell reservation activity fluctuates in this period, it is recommended to perform the SCT in a well-designed RCT to compare its efficacy with the control group to minimize the effect of confounders. Carlsson et al. conducted a trial with a good control group and found no effect on the insulin TDD and HbA1c when they matched the intervention group with the control group, while c-peptide increased in the intervention group [27].

Stem cell manufacturing cost is an important factor for evaluating the cost effectiveness of this novel intervention, but none of the eligible papers mentioned this principal point. This cost is a critical factor for promotion of the SCT to the market as a novel treatment modality.

## Conclusion

According to the results of this study, the MSCs and its combination with HSCs could be considered as “Safe Cell” for SCT in T1DM [76]. In contrast, HSC transplantation with conditioning could not be suggested as safe intervention because of potential severe adverse events and mortality.

Most of the clinical trials of SCT in T1DM were single arm. Although this meta-analysis illustrated the SCT is associated with T1DM improvement, well-designed randomized clinical trials are needed to clarify its efficacy. It is recommended to calculate the daily insulin level expressed as U/kg/day instead of measuring only daily units to help the readers in comparing the effect of SCT. There is a consensus on the c-peptide level showing that area under the curve (AUC) will better represent the beta cell function in comparison with fasting c-peptide level. The endocrinologists focus on adjusted HbA1c as a good marker for beta cell partial remission (partial remission < 9%, no remission > 11%) [4]. Therefore, for the first time, we suggested to calculate this outcome in all SCT studies for T1DM.

## Strengths and limitations

In this study, efficacy of HSC and MSC transplantation to treat the T1DM was assessed through systematic review of the trials in accordance with the MECIR [20]. Nearly all the available electronic databases and trial registries were searched from 2000 to 30 September 2019. So, it is estimated that almost all of these trials were studied in this review. Other strengths of this study include covering the applying consensus in case of conflicts, endocrinologists̓ supervision on the extracted data, presenting the inclusion and exclusion criteria related to primary studies, showing the quality effect of the primary studies on the meta-analysis results, and illustration of confidence intervals for cumulative evidence. Lack of well-designed clinical trials and control group, randomization, and blinding in almost all the studies on this topic were among the most important limitations.

## Supplementary Information

**Additional file 1.**

**Additional file 2.** “Syntaxes” which contain search strategy and search term of the 3 main databases

**Additional file 3.** Insulin TDD forest plot of all meta-analyses

**Additional file 4.** HbA1c forest plot of all meta-analyses

**Additional file 5.** C-peptide forest plot of all meta-analyses

**Additional file 6.** Adjusted HbA1c forest plot of all meta-analyses

**Additional file 7.** Authors statement

## Data Availability

All supporting data could be available.
