# Supplementary material for: Investigating the safety and efficacy of hematopoietic and mesenchymal stem cell transplantation for treatment of T1DM: a systematic review and meta-analysis
Source: Syst Rev. 2022 May 2;11:82. doi: 10.1186/s13643-022-01950-3 (PMC9059401; doi:10.1186/s13643-022-01950-3)
Supplement: Supplementary file 1 — Additional file 1. [file 13643_2022_1950_MOESM1_ESM.docx]

**Appendix 1**

**PubMed Syntax**

(("Stem Cell Transplantation"[Mesh] OR "Mesenchymal Stem Cells"[Mesh] OR "Mesenchymal Stem Cell Transplantation"[Mesh] OR "Hematopoietic Stem Cell Transplantation"[Mesh] OR (Cell[tiab] AND Stem[tiab]) OR (Mesenchymal[tiab] AND "Stromal Cell"[tiab]) OR (Cell[tiab] AND Progenitor[tiab]) OR (Transplantation[tiab] AND "Mesenchymal Stem Cell"[tiab]) OR ("Stem Cell Transplantation"[tiab] AND Mesenchymal[tiab]) OR (Transplantation[tiab] AND "Hematopoietic Stem Cell"[tiab]) OR ("Stem Cell Transplantation"[tiab] AND Hematopoietic[tiab]) OR (Transplantations[tiab] AND "Stem Cell"[tiab])) AND ("Diabetes Mellitus"[Mesh] OR ("Diabetes Mellitus"[tiab] AND "Insulin-Dependent"[tiab]) OR ("Diabetes Mellitus"[tiab] AND "Insulin Dependent"[tiab]) OR ("Diabetes Mellitus"[tiab] AND "Juvenile-Onset"[tiab]) OR ("Diabetes Mellitus"[tiab] AND "Juvenile Onset"[tiab]) OR ("Type 1"[tiab] AND "Diabetes Mellitus"[tiab]) OR ("Diabetes Mellitus"[tiab] AND "Sudden-Onset"[tiab]) OR ("Diabetes Mellitus"[tiab] AND "Sudden Onset"[tiab]) OR ("Mellitus"[tiab] AND "Sudden-Onset Diabetes"[tiab]) OR ("Diabetes Mellitus"[tiab] AND‌ "Type I"[tiab]) OR "IDDM"[tiab] OR ("Diabetes"[tiab] AND "Juvenile-Onset"[tiab]) OR ("Juvenile Onset" [tiab] AND "Diabetes"[tiab]) OR ("Diabetes Mellitus"[tiab] AND "Brittle"[tiab]) OR ("Diabetes Mellitus"[tiab] AND "Ketosis-Prone"[tiab]) OR ("Diabetes Mellitus"[tiab] AND "Ketosis Prone"[tiab]) OR (Diabetes[tiab] AND Autoimmune[tiab]))) AND 2000/01/01:2019/09/30[dp]
