# Supplementary material for: Investigating the safety and efficacy of hematopoietic and mesenchymal stem cell transplantation for treatment of T1DM: a systematic review and meta-analysis
Source: Syst Rev. 2022 May 2;11:82. doi: 10.1186/s13643-022-01950-3 (PMC9059401; doi:10.1186/s13643-022-01950-3)
Supplement: Supplementary file 2 — Additional file 2. “Syntaxes” which contain search strategy and search term of the 3 main databases [file 13643_2022_1950_MOESM2_ESM.docx]

Pubmed syntax:

| Search round | syntax | NNR | Output No. | Finded |
| --- | --- | --- | --- | --- |
| 1  Date  2016  /08/31 | (((Cell AND Stem) OR (mesenchymal AND "stromal cell") OR (Cell AND Progenitor) OR (Transplantation AND "Mesenchymal Stem Cell") OR ("Stem Cell Transplantation" AND Mesenchymal) OR (Transplantation AND "Hematopoietic Stem Cell") OR ("Stem Cell Transplantation" AND Hematopoietic) OR (Transplantations AND "Stem Cell"))  AND  (("Diabetes Mellitus" AND "Insulin-Dependent") OR ("Diabetes Mellitus" AND "Insulin Dependent") OR ("Diabetes Mellitus" AND "Juvenile-Onset") OR ("Diabetes Mellitus" AND "Juvenile Onset") OR ("Type 1" AND "Diabetes Mellitus") OR ("Diabetes Mellitus" AND "Sudden-Onset") OR ("Diabetes Mellitus" AND "Sudden Onset") OR ("Mellitus" AND "Sudden-Onset Diabetes") OR ("Diabetes Mellitus" AND "Type I") OR ("IDDM") OR ("Diabetes" AND "Juvenile-Onset") OR ("Juvenile Onset" AND "Diabetes") OR ("Diabetes Mellitus" AND "Brittle") OR ("Diabetes Mellitus" AND "Ketosis-Prone") OR ("Diabetes Mellitus" AND "Ketosis Prone") OR (Diabetes AND Autoimmune)))  AND  2000/01/01:2016/09/30[dp] | 20  For page 6 | 1192 | 503  519  553  580  584 |
| 1"  Date  2016  /09/07 | (((PRECURSOR AND CELL) OR (Cell AND Stem) OR (mesenchymal AND "stromal cell") OR (Cell AND Progenitor) OR (Transplantation AND "Mesenchymal Stem Cell") OR ("Stem Cell Transplantation" AND Mesenchymal) OR (Transplantation AND "Hematopoietic Stem Cell") OR ("Stem Cell Transplantation" AND Hematopoietic) OR (Transplantations AND "Stem Cell"))  AND  (("Diabetes Mellitus" AND "Insulin-Dependent") OR ("Diabetes Mellitus" AND "Insulin Dependent") OR ("Diabetes Mellitus" AND "Juvenile-Onset") OR ("Diabetes Mellitus" AND "Juvenile Onset") OR ("Type 1" AND "Diabetes Mellitus") OR ("Diabetes Mellitus" AND "Sudden-Onset") OR ("Diabetes Mellitus" AND "Sudden Onset") OR ("Mellitus" AND "Sudden-Onset Diabetes") OR ("Diabetes Mellitus" AND "Type I") OR ("IDDM") OR ("Diabetes" AND "Juvenile-Onset") OR ("Juvenile Onset" AND "Diabetes") OR ("Diabetes Mellitus" AND "Brittle") OR ("Diabetes Mellitus" AND "Ketosis-Prone") OR ("Diabetes Mellitus" AND "Ketosis Prone") OR (Diabetes AND Autoimmune)))  AND  2000/01/01:2016/09/30[dp] |  | 1310 |  |
| 2  Date  2016  /09/01 | (((Cell[tiab] AND Stem[tiab]) OR (mesenchymal AND "stromal cell") OR (Cell AND Progenitor) OR (Transplantation AND "Mesenchymal Stem Cell") OR ("Stem Cell Transplantation" AND Mesenchymal) OR (Transplantation AND "Hematopoietic Stem Cell") OR ("Stem Cell Transplantation" AND Hematopoietic) OR (Transplantations AND "Stem Cell"))  AND  (("Diabetes Mellitus"[tiab] AND "Insulin-Dependent"[tiab]) OR ("Diabetes Mellitus" AND "Insulin Dependent") OR ("Diabetes Mellitus" AND "Juvenile-Onset") OR ("Diabetes Mellitus" AND "Juvenile Onset") OR ("Type 1" AND "Diabetes Mellitus") OR ("Diabetes Mellitus" AND "Sudden-Onset") OR ("Diabetes Mellitus" AND "Sudden Onset") OR ("Mellitus" AND "Sudden-Onset Diabetes") OR ("Diabetes Mellitus" AND "Type I") OR ("IDDM") OR ("Diabetes" AND "Juvenile-Onset") OR ("Juvenile Onset" AND "Diabetes") OR ("Diabetes Mellitus" AND "Brittle") OR ("Diabetes Mellitus" AND "Ketosis-Prone") OR ("Diabetes Mellitus" AND "Ketosis Prone") OR (Diabetes AND Autoimmune)))  AND  2000/01/01:2016/09/30[dp] | 16.6  For page 5 | 1005 | 408  424  438  465  485  489 |
| 3  Date  2016  /09/01 | (((Cell[tiab] AND Stem[tiab]) OR (mesenchymal[tiab] AND "stromal cell"[tiab]) OR (Cell AND Progenitor) OR (Transplantation AND "Mesenchymal Stem Cell") OR ("Stem Cell Transplantation" AND Mesenchymal) OR (Transplantation AND "Hematopoietic Stem Cell") OR ("Stem Cell Transplantation" AND Hematopoietic) OR (Transplantations AND "Stem Cell"))  AND  (("Diabetes Mellitus"[tiab] AND "Insulin-Dependent"[tiab]) OR ("Diabetes Mellitus"[tiab] AND "Insulin Dependent"[tiab]) OR ("Diabetes Mellitus" AND "Juvenile-Onset") OR ("Diabetes Mellitus" AND "Juvenile Onset") OR ("Type 1" AND "Diabetes Mellitus") OR ("Diabetes Mellitus" AND "Sudden-Onset") OR ("Diabetes Mellitus" AND "Sudden Onset") OR ("Mellitus" AND "Sudden-Onset Diabetes") OR ("Diabetes Mellitus" AND "Type I") OR ("IDDM") OR ("Diabetes" AND "Juvenile-Onset") OR ("Juvenile Onset" AND "Diabetes") OR ("Diabetes Mellitus" AND "Brittle") OR ("Diabetes Mellitus" AND "Ketosis-Prone") OR ("Diabetes Mellitus" AND "Ketosis Prone") OR (Diabetes AND Autoimmune)))  AND  2000/01/01:2016/09/30[dp] |  | 999 |  |
| 4  Date  2016  /09/01 | (((Cell[tiab] AND Stem[tiab]) OR (mesenchymal[tiab] AND "stromal cell"[tiab]) OR (Cell[tiab] AND Progenitor[tiab]) OR (Transplantation[tiab] AND "Mesenchymal Stem Cell"[tiab]) OR ("Stem Cell Transplantation"[tiab] AND Mesenchymal[tiab]) OR (Transplantation AND "Hematopoietic Stem Cell") OR ("Stem Cell Transplantation" AND Hematopoietic) OR (Transplantations AND "Stem Cell"))  AND  (("Diabetes Mellitus"[tiab] AND "Insulin-Dependent"[tiab]) OR ("Diabetes Mellitus"[tiab] AND "Insulin Dependent"[tiab]) OR ("Diabetes Mellitus"[tiab] AND "Juvenile-Onset"[tiab]) OR ("Diabetes Mellitus"[tiab] AND "Juvenile Onset"[tiab]) OR ("Type 1" AND "Diabetes Mellitus") OR ("Diabetes Mellitus" AND "Sudden-Onset") OR ("Diabetes Mellitus" AND "Sudden Onset") OR ("Mellitus" AND "Sudden-Onset Diabetes") OR ("Diabetes Mellitus" AND "Type I") OR ("IDDM") OR ("Diabetes" AND "Juvenile-Onset") OR ("Juvenile Onset" AND "Diabetes") OR ("Diabetes Mellitus" AND "Brittle") OR ("Diabetes Mellitus" AND "Ketosis-Prone") OR ("Diabetes Mellitus" AND "Ketosis Prone") OR (Diabetes AND Autoimmune)))  AND  2000/01/01:2016/09/30[dp] | 13.3  For pages 4&5 | 938 | 315  317  318  336  343  346  359  370  373  380  396  408  433  451  455 |
| 4"  Date  2016  /09/07 | (((Cell[tiab] AND Stem[tiab]) OR (mesenchymal[tiab] AND "stromal cell"[tiab]) OR (Cell[tiab] AND Progenitor[tiab]) OR (Transplantation[tiab] AND "Mesenchymal Stem Cell"[tiab]) OR ("Stem Cell Transplantation"[tiab] AND Mesenchymal[tiab]) OR (precursor[tiab] AND cell[tiab]) OR (Transplantation AND "Hematopoietic Stem Cell") OR ("Stem Cell Transplantation" AND Hematopoietic) OR (Transplantations AND "Stem Cell"))  AND  (("Diabetes Mellitus"[tiab] AND "Insulin-Dependent"[tiab]) OR ("Diabetes Mellitus"[tiab] AND "Insulin Dependent"[tiab]) OR ("Diabetes Mellitus"[tiab] AND "Juvenile-Onset"[tiab]) OR ("Diabetes Mellitus"[tiab] AND "Juvenile Onset"[tiab]) OR ("Type 1" AND "Diabetes Mellitus") OR ("Diabetes Mellitus" AND "Sudden-Onset") OR ("Diabetes Mellitus" AND "Sudden Onset") OR ("Mellitus" AND "Sudden-Onset Diabetes") OR ("Diabetes Mellitus" AND "Type I") OR ("IDDM") OR ("Diabetes" AND "Juvenile-Onset") OR ("Juvenile Onset" AND "Diabetes") OR ("Diabetes Mellitus" AND "Brittle") OR ("Diabetes Mellitus" AND "Ketosis-Prone") OR ("Diabetes Mellitus" AND "Ketosis Prone") OR (Diabetes AND Autoimmune)))  AND  2000/01/01:2016/09/30[dp] | 15.5 | 1005 | 361  364  388  394  415  427  453  474  478 |
| 5  8/20/2017 | (((Cell[tiab] AND Stem[tiab]) OR (mesenchymal[tiab] AND "stromal cell"[tiab]) OR (Cell[tiab] AND Progenitor[tiab]) OR (Transplantation[tiab] AND "Mesenchymal Stem Cell"[tiab]) OR ("Stem Cell Transplantation"[tiab] AND Mesenchymal[tiab]) OR (precursor[tiab] AND cell[tiab]) OR (Transplantation AND "Hematopoietic Stem Cell") OR ("Stem Cell Transplantation" AND Hematopoietic) OR (Transplantations AND "Stem Cell"))  AND  (("Diabetes Mellitus"[tiab] AND "Insulin-Dependent"[tiab]) OR ("Diabetes Mellitus"[tiab] AND "Insulin Dependent"[tiab]) OR ("Diabetes Mellitus"[tiab] AND "Juvenile-Onset"[tiab]) OR ("Diabetes Mellitus"[tiab] AND "Juvenile Onset"[tiab]) OR ("Type 1" AND "Diabetes Mellitus") OR ("Diabetes Mellitus" AND "Sudden-Onset") OR ("Diabetes Mellitus" AND "Sudden Onset") OR ("Mellitus" AND "Sudden-Onset Diabetes") OR ("Diabetes Mellitus" AND "Type I") OR ("IDDM") OR ("Diabetes" AND "Juvenile-Onset") OR ("Juvenile Onset" AND "Diabetes") OR ("Diabetes Mellitus" AND "Brittle") OR ("Diabetes Mellitus" AND "Ketosis-Prone") OR ("Diabetes Mellitus" AND "Ketosis Prone") OR (Diabetes AND Autoimmune)))  AND  2000/01/01:2019/09/31[dp] |  |  |  |

Scopus syntax:

| Search round | Syntax | NNR | Result No. | Finded |
| --- | --- | --- | --- | --- |
| 1  Date  2016/  09/11 | (((TITLE-ABS(Cell) AND TITLE-ABS(Stem)) OR (TITLE-ABS(mesenchymal) AND TITLE-ABS("stromal cell")) OR (TITLE-ABS(Cell) AND TITLE-ABS(Progenitor)) OR (TITLE-ABS(Transplantation) AND TITLE-ABS("Mesenchymal Stem Cell")) OR (TITLE-ABS("Stem Cell Transplantation") AND TITLE-ABS(Mesenchymal)) OR (TITLE-ABS(precursor) AND TITLE-ABS(cell)) (ALL(Transplantation) AND ALL("Hematopoietic Stem Cell")) OR (ALL("Stem Cell Transplantation") AND ALL(Hematopoietic)) OR (ALL(Transplantations) AND ALL("Stem Cell")))  AND  ((TITLE-ABS("Diabetes Mellitus") AND TITLE-ABS("Insulin-Dependent")) OR (TITLE-ABS("Diabetes Mellitus") AND TITLE-ABS("Insulin Dependent")) OR (TITLE-ABS("Diabetes Mellitus") AND TITLE-ABS("Juvenile-Onset")) OR (TITLE-ABS("Diabetes Mellitus") AND TITLE-ABS("Juvenile Onset")) OR (ALL("Type 1") AND ALL("Diabetes Mellitus")) OR (ALL("Diabetes Mellitus") AND ALL("Sudden-Onset")) OR (ALL("Diabetes Mellitus") AND ALL("Sudden Onset")) OR (ALL("Mellitus") AND ALL("Sudden-Onset Diabetes")) OR (ALL("Diabetes Mellitus") AND ALL("Type I")) OR ALL("IDDM") OR (ALL("Diabetes") AND ALL("Juvenile-Onset")) OR (ALL("Juvenile Onset") AND ALL("Diabetes")) OR (ALL("Diabetes Mellitus") AND ALL("Brittle")) OR (ALL("Diabetes Mellitus") AND ALL("Ketosis-Prone")) OR (ALL("Diabetes Mellitus") AND ALL("Ketosis Prone")) OR (ALL(Diabetes) AND ALL(Autoimmune)))) AND PUBYEAR AFT 1999 | 50 | 4546 | 1901  1972 |
| 2  Date  2016/  09/11 | ( ( ( TITLE-ABS ( cell )  AND  TITLE-ABS ( stem ) )  OR  ( TITLE-ABS ( mesenchymal )  AND  TITLE-ABS ( "stromal cell" ) )  OR  ( TITLE-ABS ( cell )  AND  TITLE-ABS ( progenitor ) )  OR  ( TITLE-ABS ( transplantation )  AND  TITLE-ABS ( "Mesenchymal Stem Cell" ) )  OR  ( TITLE-ABS ( "Stem Cell Transplantation" )  AND  TITLE-ABS ( mesenchymal ) )  OR  ( TITLE-ABS ( precursor )  AND  TITLE-ABS ( cell ) )  ( TITLE-ABS ( transplantation )  AND  TITLE-ABS ( "Hematopoietic Stem Cell" ) )  OR  ( TITLE-ABS ( "Stem Cell Transplantation" )  AND  TITLE-ABS ( hematopoietic ) )  OR  ( ALL ( transplantations )  AND  TITLE-ABS ( "Stem Cell" ) ) )  AND  ( ( TITLE-ABS ( "Diabetes Mellitus" )  AND  TITLE-ABS ( "Insulin-Dependent" ) )  OR  ( TITLE-ABS ( "Diabetes Mellitus" )  AND  TITLE-ABS ( "Insulin Dependent" ) )  OR  ( TITLE-ABS ( "Diabetes Mellitus" )  AND  TITLE-ABS ( "Juvenile-Onset" ) )  OR  ( TITLE-ABS ( "Diabetes Mellitus" )  AND  TITLE-ABS ( "Juvenile Onset" ) )  OR  ( ALL ( "Type 1" )  AND  ALL ( "Diabetes Mellitus" ) )  OR  ( ALL ( "Diabetes Mellitus" )  AND  ALL ( "Sudden-Onset" ) )  OR  ( ALL ( "Diabetes Mellitus" )  AND  ALL ( "Sudden Onset" ) )  OR  ( ALL ( "Mellitus" )  AND  ALL ( "Sudden-Onset Diabetes" ) )  OR  ( ALL ( "Diabetes Mellitus" )  AND  ALL ( "Type I" ) )  OR  ALL ( "IDDM" )  OR  ( ALL ( "Diabetes" )  AND  ALL ( "Juvenile-Onset" ) )  OR  ( ALL ( "Juvenile Onset" )  AND  ALL ( "Diabetes" ) )  OR  ( ALL ( "Diabetes Mellitus" )  AND  ALL ( "Brittle" ) )  OR  ( ALL ( "Diabetes Mellitus" )  AND  ALL ( "Ketosis-Prone" ) )  OR  ( ALL ( "Diabetes Mellitus" )  AND  ALL ( "Ketosis Prone" ) )  OR ( ALL ( diabetes )  AND  ALL ( autoimmune ) ) ) )  AND  PUBYEAR  >  1999 | 100 | 3649 | 1546 |
| 3  Date  2016/  09/11 | ( ( ( TITLE-ABS ( cell )  AND  TITLE-ABS ( stem ) )  OR  ( TITLE-ABS ( mesenchymal )  AND  TITLE-ABS ( "stromal cell" ) )  OR  ( TITLE-ABS ( cell )  AND  TITLE-ABS ( progenitor ) )  OR  ( TITLE-ABS ( transplantation )  AND  TITLE-ABS ( "Mesenchymal Stem Cell" ) )  OR  ( TITLE-ABS ( "Stem Cell Transplantation" )  AND  TITLE-ABS ( mesenchymal ) )  OR  ( TITLE-ABS ( precursor )  AND  TITLE-ABS ( cell ) )  ( TITLE-ABS ( transplantation )  AND  TITLE-ABS ( "Hematopoietic Stem Cell" ) )  OR  ( TITLE-ABS ( "Stem Cell Transplantation" )  AND  TITLE-ABS ( hematopoietic ) )  OR  ( ALL ( transplantations )  AND  TITLE-ABS ( "Stem Cell" ) ) )  AND  ( ( TITLE-ABS ( "Diabetes Mellitus" )  AND  TITLE-ABS ( "Insulin-Dependent" ) )  OR  ( TITLE-ABS ( "Diabetes Mellitus" )  AND  TITLE-ABS ( "Insulin Dependent" ) )  OR  ( TITLE-ABS ( "Diabetes Mellitus" )  AND  TITLE-ABS ( "Juvenile-Onset" ) )  OR  ( TITLE-ABS ( "Diabetes Mellitus" )  AND  TITLE-ABS ( "Juvenile Onset" ) )  OR  ( ALL ( "Type 1" )  AND  TITLE-ABS ( "Diabetes Mellitus" ) )  OR  ( ALL ( "Diabetes Mellitus" )  AND  ALL ( "Sudden-Onset" ) )  OR  ( ALL ( "Diabetes Mellitus" )  AND  ALL ( "Sudden Onset" ) )  OR  ( ALL ( "Mellitus" )  AND  ALL ( "Sudden-Onset Diabetes" ) )  OR  ( ALL ( "Diabetes Mellitus" )  AND  ALL ( "Type I" ) )  OR  ALL ( "IDDM" )  OR  ( ALL ( "Diabetes" )  AND  ALL ( "Juvenile-Onset" ) )  OR  ( ALL ( "Juvenile Onset" )  AND  ALL ( "Diabetes" ) )  OR  ( ALL ( "Diabetes Mellitus" )  AND  ALL ( "Brittle" ) )  OR  ( ALL ( "Diabetes Mellitus" )  AND  ALL ( "Ketosis-Prone" ) )  OR  ( ALL ( "Diabetes Mellitus" )  AND  ALL ( "Ketosis Prone" ) )  OR  ( ALL ( diabetes )  AND  ALL ( autoimmune ) ) ) )  AND  PUBYEAR  >  1999 | 50 | 2806 | 1324  1389 |
| 4  Date  2016/  09/11 | ( ( ( TITLE-ABS ( cell )  AND  TITLE-ABS ( stem ) )  OR  ( TITLE-ABS ( mesenchymal )  AND  TITLE-ABS ( "stromal cell" ) )  OR  ( TITLE-ABS ( cell )  AND  TITLE-ABS ( progenitor ) )  OR  ( TITLE-ABS ( transplantation )  AND  TITLE-ABS ( "Mesenchymal Stem Cell" ) )  OR  ( TITLE-ABS ( "Stem Cell Transplantation" )  AND  TITLE-ABS ( mesenchymal ) )  OR  ( TITLE-ABS ( precursor )  AND  TITLE-ABS ( cell ) )  ( TITLE-ABS ( transplantation )  AND  TITLE-ABS ( "Hematopoietic Stem Cell" ) )  OR  ( TITLE-ABS ( "Stem Cell Transplantation" )  AND  TITLE-ABS ( hematopoietic ) )  OR  ( ALL ( transplantations )  AND  TITLE-ABS ( "Stem Cell" ) ) )  AND  ( ( TITLE-ABS ( "Diabetes Mellitus" )  AND  TITLE-ABS ( "Insulin-Dependent" ) )  OR  ( TITLE-ABS ( "Diabetes Mellitus" )  AND  TITLE-ABS ( "Insulin Dependent" ) )  OR  ( TITLE-ABS ( "Diabetes Mellitus" )  AND  TITLE-ABS ( "Juvenile-Onset" ) )  OR  ( TITLE-ABS ( "Diabetes Mellitus" )  AND  TITLE-ABS ( "Juvenile Onset" ) )  OR  ( TITLE-ABS ( "Type 1" )  AND  TITLE-ABS ( "Diabetes Mellitus" ) )  OR  ( TITLE-ABS ( "Diabetes Mellitus" )  AND  TITLE-ABS ( "Sudden-Onset" ) )  OR  ( TITLE-ABS ( "Diabetes Mellitus" )  AND  TITLE-ABS ( "Sudden Onset" ) )  OR  ( TITLE-ABS ( "Mellitus" )  AND  TITLE-ABS ( "Sudden-Onset Diabetes" ) )  OR  ( TITLE-ABS ( "Diabetes Mellitus" )  AND  TITLE-ABS ( "Type I" ) )  OR  TITLE-ABS ( "IDDM" )  OR  ( ALL ( "Diabetes" )  AND  ALL ( "Juvenile-Onset" ) )  OR  ( ALL ( "Juvenile Onset" )  AND  ALL ( "Diabetes" ) )  OR  ( ALL ( "Diabetes Mellitus" )  AND  ALL ( "Brittle" ) )  OR  ( ALL ( "Diabetes Mellitus" )  AND  ALL ( "Ketosis-Prone" ) )  OR  ( ALL ( "Diabetes Mellitus" )  AND  ALL ( "Ketosis Prone" ) )  OR  ( ALL ( diabetes )  AND  ALL ( autoimmune ) ) ) )  AND  PUBYEAR  >  1999 | 33 | 2273 | 1023  1037  1069 |
| 5  Date  2016/  09/17 | ( ( ( TITLE-ABS ( cell )  AND  TITLE-ABS ( stem ) )  OR  ( TITLE-ABS ( mesenchymal )  AND  TITLE-ABS ( "stromal cell" ) )  OR  ( TITLE-ABS ( cell )  AND  TITLE-ABS ( progenitor ) )  OR  ( TITLE-ABS ( transplantation )  AND  TITLE-ABS ( "Mesenchymal Stem Cell" ) )  OR  ( TITLE-ABS ( "Stem Cell Transplantation" )  AND  TITLE-ABS ( mesenchymal ) )  OR  ( TITLE-ABS ( precursor )  AND  TITLE-ABS ( cell ) ) OR ( TITLE-ABS ( transplantation )  AND  TITLE-ABS ( "Hematopoietic Stem Cell" ) )  OR  ( TITLE-ABS ( "Stem Cell Transplantation" )  AND  TITLE-ABS ( hematopoietic ) )  OR  ( ALL ( transplantations )  AND  TITLE-ABS ( "Stem Cell" ) ) )  AND  ( ( TITLE-ABS ( "Diabetes Mellitus" )  AND  TITLE-ABS ( "Insulin-Dependent" ) )  OR  ( TITLE-ABS ( "Diabetes Mellitus" )  AND  TITLE-ABS ( "Insulin Dependent" ) )  OR  ( TITLE-ABS ( "Diabetes Mellitus" )  AND  TITLE-ABS ( "Juvenile-Onset" ) )  OR  ( TITLE-ABS ( "Diabetes Mellitus" )  AND  TITLE-ABS ( "Juvenile Onset" ) )  OR  ( TITLE-ABS ( "Type 1" )  AND  TITLE-ABS ( "Diabetes Mellitus" ) )  OR  ( TITLE-ABS ( "Diabetes Mellitus" )  AND  TITLE-ABS ( "Sudden-Onset" ) )  OR  ( TITLE-ABS ( "Diabetes Mellitus" )  AND  TITLE-ABS ( "Sudden Onset" ) )  OR  ( TITLE-ABS ( "Mellitus" )  AND  TITLE-ABS ( "Sudden-Onset Diabetes" ) )  OR  ( TITLE-ABS ( "Diabetes Mellitus" )  AND  TITLE-ABS ( "Type I" ) )  OR  TITLE-ABS ( "IDDM" )  OR  ( TITLE-ABS ( "Diabetes" )  AND  TITLE-ABS ( "Juvenile-Onset" ) )  OR  ( TITLE-ABS ( "Juvenile Onset" )  AND  TITLE-ABS ( "Diabetes" ) )  OR  ( TITLE-ABS ( "Diabetes Mellitus" )  AND  TITLE-ABS ( "Brittle" ) )  OR  ( TITLE-ABS ( "Diabetes Mellitus" )  AND  TITLE-ABS ( "Ketosis-Prone" ) )  OR  ( TITLE-ABS ( "Diabetes Mellitus" )  AND  TITLE-ABS ( "Ketosis Prone" ) )  OR  ( ALL ( diabetes )  AND  ALL ( autoimmune ) ) ) )  AND  PUBYEAR  >  1999 | 33 | 2266 | 1022  1036  1068 |
| 6  8/20/2017 | ))TITLE-ABS-KEY(cell) AND TITLE-ABS-KEY(stem)) OR (TITLE-ABS-KEY(mesenchymal) AND TITLE-ABS-KEY(stromal cell)) OR (TITLE-ABS-KEY(cell) AND TITLE-ABS-KEY(progenitor)) OR (TITLE-ABS-KEY(transplantation) AND TITLE-ABS-KEY(Mesenchymal Stem Cell)) OR (TITLE-ABS-KEY(Stem Cell Transplantation) AND TITLE-ABS-KEY(mesenchymal)) OR (TITLE-ABS-KEY(precursor) AND TITLE-ABS-KEY(cell)) OR (TITLE-ABS-KEY(transplantation) AND TITLE-ABS-KEY(Hematopoietic Stem Cell)) OR (TITLE-ABS-KEY(Stem Cell Transplantation) AND TITLE-ABS-KEY(hematopoietic)) OR (ALL(transplantations) AND TITLE-ABS-KEY(Stem Cell))) AND ((TITLE-ABS-KEY(Diabetes Mellitus) AND TITLE-ABS-KEY(Insulin-Dependent)) OR (TITLE-ABS-KEY(Diabetes Mellitus) AND TITLE-ABS-KEY(Insulin Dependent)) OR (TITLE-ABS-KEY(Diabetes Mellitus) AND TITLE-ABS-KEY(Juvenile-Onset)) OR (TITLE-ABS-KEY(Diabetes Mellitus) AND TITLE-ABS-KEY(Juvenile Onset)) OR (TITLE-ABS-KEY(Type 1) AND TITLE-ABS-KEY(Diabetes Mellitus)) OR (TITLE-ABS-KEY(Diabetes Mellitus) AND TITLE-ABS-KEY(Sudden-Onset)) OR (TITLE-ABS-KEY(Diabetes Mellitus) AND TITLE-ABS-KEY(Sudden Onset)) OR (TITLE-ABS-KEY(Mellitus) AND TITLE-ABS-KEY(Sudden-Onset Diabetes)) OR (TITLE-ABS-KEY(Diabetes Mellitus) AND TITLE-ABS-KEY(Type I)) OR TITLE-ABS-KEY(IDDM) OR (TITLE-ABS-KEY(Diabetes) AND TITLE-ABS-KEY(Juvenile-Onset)) OR (TITLE-ABS-KEY(Juvenile Onset) AND TITLE-ABS-KEY(Diabetes)) OR (TITLE-ABS-KEY(Diabetes Mellitus) AND TITLE-ABS-KEY(Brittle)) OR (TITLE-ABS-KEY(Diabetes Mellitus) AND TITLE-ABS-KEY(Ketosis-Prone)) OR (TITLE-ABS-KEY(Diabetes Mellitus) AND TITLE-ABS-KEY(Ketosis Prone)) OR (ALL (diabetes) AND ALL (autoimmune)))  AND  PUBYEAR  >  1999 |  |  |  |

ISI

| Search round | Syntax | NNR | Result No. | Finded |
| --- | --- | --- | --- | --- |
| 1  Date  2016/  09/19 | ((TS=(Cell AND Stem) OR TS=(mesenchymal AND ("stromal cell")) OR TS=(Cell AND Progenitor) OR TS=(Transplantation AND ("Mesenchymal Stem Cell")) OR TS=(("Stem Cell Transplantation") AND Mesenchymal) OR TS=(precursor AND cell) TS=(Transplantation AND ("Hematopoietic Stem Cell")) OR TS=(("Stem Cell Transplantation") AND Hematopoietic) OR TS=(Transplantations AND ("Stem Cell")))  AND  (TS=(("Diabetes Mellitus") AND ("Insulin-Dependent")) OR TS=(("Diabetes Mellitus") AND ("Insulin Dependent")) OR TS=(("Diabetes Mellitus") AND ("Juvenile-Onset")) OR TS=(("Diabetes Mellitus") AND ("Juvenile Onset")) OR TS=(("Type 1") AND ("Diabetes Mellitus")) OR TS=(("Diabetes Mellitus") AND ("Sudden-Onset")) OR TS=(("Diabetes Mellitus") AND ("Sudden Onset")) OR TS=(Mellitus AND ("Sudden-Onset Diabetes")) OR TS=(("Diabetes Mellitus") AND ("Type I")) OR TS=("IDDM") OR TS=(Diabetes AND ("Juvenile-Onset")) OR TS=(("Juvenile Onset") AND Diabetes) OR TS=(("Diabetes Mellitus") AND Brittle) OR TS=(("Diabetes Mellitus") AND ("Ketosis-Prone")) OR TS=(("Diabetes Mellitus") AND ("Ketosis Prone")) OR TS=(Diabetes AND Autoimmune)))  AND  PY(2000-2016) |  |  |  |
|  |  |  |  |  |

.

**The final edited syntax of three database:**

**PubMed**

(("Stem Cell Transplantation"[Mesh] OR "Mesenchymal Stem Cells"[Mesh] OR "Mesenchymal Stem Cell Transplantation"[Mesh] OR"Hematopoietic Stem Cell Transplantation"[Mesh] OR (Cell[tiab] AND Stem[tiab]) OR (mesenchymal[tiab] AND "stromal cell"[tiab]) OR (Cell[tiab] AND Progenitor[tiab]) OR (Transplantation[tiab] AND"Mesenchymal Stem Cell"[tiab]) OR ("Stem Cell Transplantation"[tiab] AND Mesenchymal[tiab]) OR (Transplantation[tiab] AND"Hematopoietic Stem Cell"[tiab]) OR ("Stem Cell Transplantation"[tiab] AND Hematopoietic[tiab]) OR (Transplantations[tiab] AND"Stem Cell"[tiab])) AND ("Diabetes Mellitus"[Mesh] OR ("Diabetes Mellitus"[tiab] AND"Insulin-Dependent"[tiab]) OR ("Diabetes Mellitus"[tiab] AND"Insulin Dependent"[tiab]) OR ("Diabetes Mellitus"[tiab] AND"Juvenile-Onset"[tiab]) OR ("Diabetes Mellitus"[tiab] AND "Juvenile Onset"[tiab]) OR ("Type 1"[tiab] AND"Diabetes Mellitus"[tiab]) OR ("Diabetes Mellitus"[tiab] AND"Sudden-Onset"[tiab]) OR ("Diabetes Mellitus"[tiab] AND"Sudden Onset"[tiab]) OR ("Mellitus"[tiab] AND"Sudden-Onset Diabetes"[tiab]) OR ("Diabetes Mellitus"[tiab] AND"Type I"[tiab]) OR "IDDM"[tiab] OR ("Diabetes"[tiab] AND "Juvenile-Onset"[tiab]) OR ("Juvenile Onset" [tiab] AND"Diabetes"[tiab]) OR ("Diabetes Mellitus"[tiab] AND"Brittle"[tiab]) OR ("Diabetes Mellitus"[tiab] AND"Ketosis-Prone"[tiab]) OR ("Diabetes Mellitus"[tiab] AND"Ketosis Prone"[tiab]) OR (Diabetes[tiab] AND Autoimmune[tiab]))) AND 2000/01/01:2019/09/30[dp]

**Scopus**

TITLE-ABS-KEY( (((CellAND Stem) OR (mesenchymal AND "stromal cell") OR (Cell AND Progenitor) OR (Transplantation AND"Mesenchymal Stem Cell") OR ("Stem Cell Transplantation" AND Mesenchymal) OR (Transplantation AND"Hematopoietic Stem Cell") OR ("Stem Cell Transplantation" AND Hematopoietic) OR (Transplantations AND"Stem Cell")) AND (("Diabetes Mellitus" AND"Insulin-Dependent") OR ("Diabetes Mellitus" AND"Insulin Dependent") OR ("Diabetes Mellitus" AND"Juvenile-Onset") OR ("Diabetes Mellitus" AND "Juvenile Onset") OR ("Type 1" AND"Diabetes Mellitus") OR ("Diabetes Mellitus" AND"Sudden-Onset") OR ("Diabetes Mellitus" AND"Sudden Onset") OR ("Mellitus" AND"Sudden-Onset Diabetes") OR ("Diabetes Mellitus" AND"Type I") OR ("IDDM") OR ("Diabetes" AND "Juvenile-Onset") OR ("Juvenile Onset" AND"Diabetes") OR ("Diabetes Mellitus" AND"Brittle") OR ("Diabetes Mellitus" AND"Ketosis-Prone") OR ("Diabetes Mellitus" AND"Ketosis Prone") OR (Diabetes AND Autoimmune))) ) AND  PUBYEAR  >  2019

**ISI**

TS=((((CellAND Stem) OR (mesenchymal AND "stromal cell") OR (Cell AND Progenitor) OR (Transplantation AND"Mesenchymal Stem Cell") OR ("Stem Cell Transplantation" AND Mesenchymal) OR (Transplantation AND "Hematopoietic Stem Cell") OR ("Stem Cell Transplantation" AND Hematopoietic) OR (Transplantations AND "Stem Cell")) AND (("Diabetes Mellitus" AND "Insulin-Dependent") OR ("Diabetes Mellitus" AND "Insulin Dependent") OR ("Diabetes Mellitus" AND "Juvenile-Onset") OR ("Diabetes Mellitus" AND "Juvenile Onset") OR ("Type 1" AND "Diabetes Mellitus") OR ("Diabetes Mellitus" AND "Sudden-Onset") OR ("Diabetes Mellitus" AND "Sudden Onset") OR ("Mellitus" AND "Sudden-Onset Diabetes") OR ("Diabetes Mellitus" AND "Type I") OR ("IDDM") OR ("Diabetes" AND "Juvenile-Onset") OR ("Juvenile Onset" AND "Diabetes") OR ("Diabetes Mellitus" AND "Brittle") OR ("Diabetes Mellitus" AND "Ketosis-Prone") OR ("Diabetes Mellitus" AND "Ketosis Prone") OR (Diabetes AND Autoimmune))) ) AND PY=(2000-2019)
