# Supplementary material for: Investigating the safety and efficacy of hematopoietic and mesenchymal stem cell transplantation for treatment of T1DM: a systematic review and meta-analysis
Source: Syst Rev. 2022 May 2;11:82. doi: 10.1186/s13643-022-01950-3 (PMC9059401; doi:10.1186/s13643-022-01950-3)
Supplement: Supplementary file 7 — Additional file 7. Authors statement [file 13643_2022_1950_MOESM7_ESM.docx]

## Statement of Authorship

The submitting author affirms that all individuals listed as authors have met the criteria of authorship. In order to meet the requirements of authorship, each author must have contributed to at least one aspect of each of the four criteria, as listed below. Please note that for Criteria 1 and 2, authors need only to meet one of the two items listed. Any individuals not meeting the criteria have been mentioned in the Acknowledgements section of the manuscript.

Per the criteria defined by the [International Committee for Medical Journal Editors](http://www.icmje.org/recommendations/browse/roles-and-responsibilities/defining-the-role-of-authors-and-contributors.html) (ICJME), please note the contribution made by each author listed in the manuscript. **Please type each role into the boxes that apply.**

| Author (Last name, First Initial)  e.g. Smith, J | **Criteria 1** | | **Criteria 2** | | **Criteria 3** | **Criteria 4** |
| --- | --- | --- | --- | --- | --- | --- |
|  | contributed to conception or design | contributed to acquisition, analysis, or interpretation | drafted the manuscript | critically revised the manuscript | gave final approval | Agrees to be accountable for all aspects of work ensuring integrity and accuracy |
| Madani, S | Contributed to conception and design | Contributed to analysis | Drafted the manuscript | critically revised the manuscript | Gave final approval | Agrees to be accountable for all aspects of work ensuring integrity and accuracy |
| Larijani, B | contributed to conception and design | contributed to acquisition, analysis, and interpretation | Drafted the manuscript | critically revised the manuscript | gave final approval | Agrees to be accountable for all aspects of work ensuring integrity and accuracy |
| Aghayan, HR | contributed to conception and design | contributed to acquisition, analysis, and interpretation | Drafted the manuscript |  | gave final approval | Agrees to be accountable for all aspects of work ensuring integrity and accuracy |
| Rezaie, N | contributed to conception and design | contributed to acquisition, analysis, and interpretation | Drafted the manuscript | critically revised the manuscript | gave final approval | Agrees to be accountable for all aspects of work ensuring integrity and accuracy |
| Setudeh,A | contributed to conception and design |  | Drafted the manuscript |  | gave final approval | Agrees to be accountable for all aspects of work ensuring integrity and accuracy |
| Rouhifard, M | contributed to conception and design |  | Drafted the manuscript |  | gave final approval | Agrees to be accountable for all aspects of work ensuring integrity and accuracy |
| Amanzadi, M | contributed to conception and design | contributed to acquisition, analysis, and interpretation | Drafted the manuscript |  | gave final approval | Agrees to be accountable for all aspects of work ensuring integrity and accuracy |
